# Supplementary material for: Stomatin modulates adipogenesis through the ERK pathway and regulates fatty acid uptake and lipid droplet growth
Source: Nat Commun. 2022 Jul 19;13:4174. doi: 10.1038/s41467-022-31825-z (PMC9296665; doi:10.1038/s41467-022-31825-z)
Supplement: Supplementary file 1 — Supplementary Information [file 41467_2022_31825_MOESM1_ESM.pdf]

Supplementary information

Stomatin modulates adipogenesis through the ERK pathway and
regulates fatty acid uptake and lipid droplet growth

Shao-Chin Wu[1], Yuan-Ming Lo[2], Jui-Hao Lee[3], Chin-Yau Chen[4], Tung-Wei Chen[1], Hong-Wen Liu[5], Wei-Nan Lian[2], Kate Hua [6], Chen-Chung Liao[6,7], Wei-Ju Lin [6], Chih-Yung Yang[8], Chien-Yi Tung[6#], Chi-Hung Lin[1,2,6,9*]

1. Institute of Biophotonics, National Yang Ming Chiao Tung University, Taipei, Taiwan
2. Institute of Microbiology and Immunology, National Yang Ming Chiao Tung University, Taipei, Taiwan
3. Taiwan International Graduate Program in Molecular Medicine, National Yang Ming Chiao Tung University and Academia Sinica, Taipei, Taiwan
4. Department of Surgery, National Yang Ming Chiao Tung University Hospital, I-Lan, Taiwan
5. Chong Hin Loon Memorial Cancer and Biotherapy Research Center, National Yang Ming Chiao Tung University, Taipei, Taiwan
6. Genomics Center for Clinical and Biotechnological Applications, Cancer Progression Research Center, National Yang Ming Chiao Tung University, Taipei, Taiwan
7. Metabolomics-Proteomics Research Center, National Yang Ming Chiao Tung University, Taipei, Taiwan
8. Department of Education and Research, Taipei City Hospital, Taipei, Taiwan
9. Department of Biological Science and Technology, National Yang Ming Chiao Tung University, Hsinchu, Taiwan

Supplementary Figure 1-8
Supplementary Table 1

a.

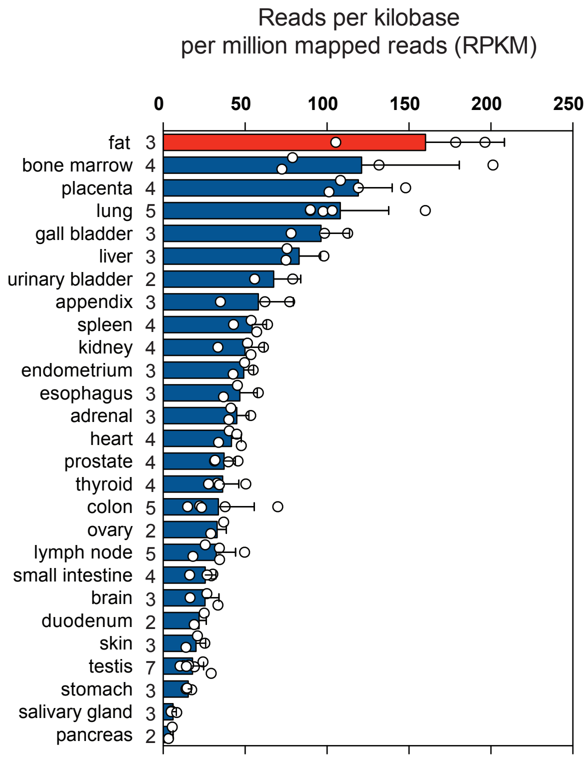

b.

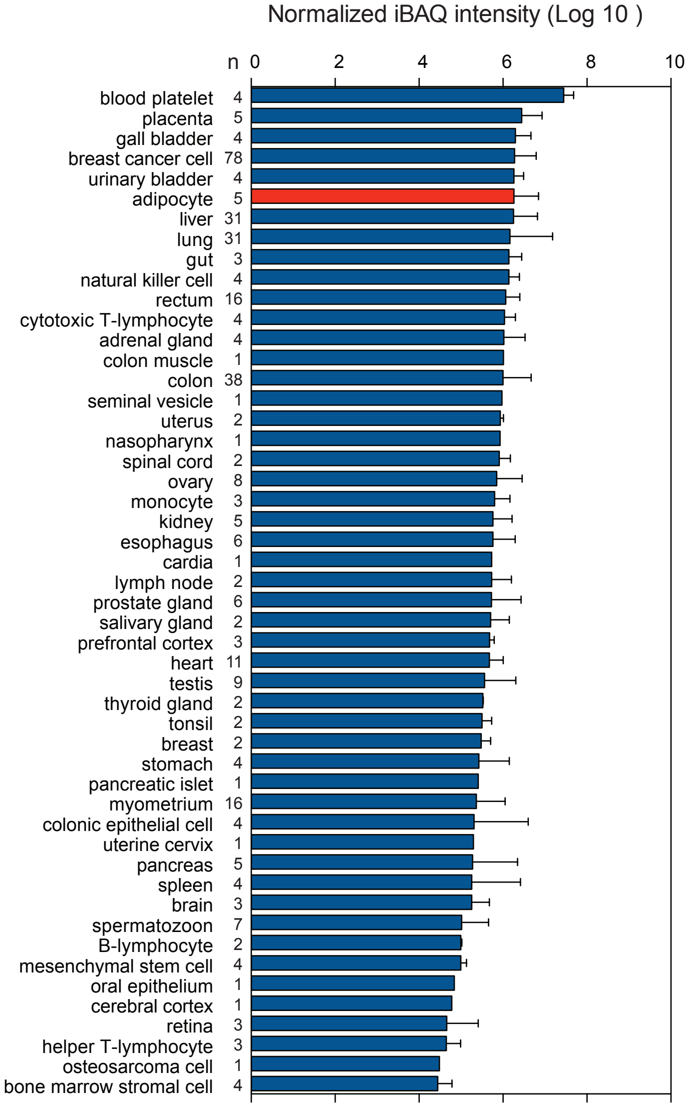

Supplementary Figure 1: In silico analyses of stomatin mRNA and proteins in different human tissues.
a. The bar charts were reproduced based on the NCBI database: "https://www.ncbi.nlm.nih.gov/gene/2040?report=expression&bioproject=PRJEB4337", containing RNA sequencing of total RNA from 95 human tissues. Adipose tissues express the highest amount of mRNA among all tissues examined. Mean ± s.d. is shown. Each dot represents one person. **b.** The bar charts were reproduced using ProteomicsDB: "https://www.proteomicsdb.org/proteomicsdb/#protein/proteinDetails/54374/expressiqqon". Mean with error bar. The error bars indicate the lowest and highest abundance level for stomatin proteins in different human tissues. Source data are provided as a Source data file.

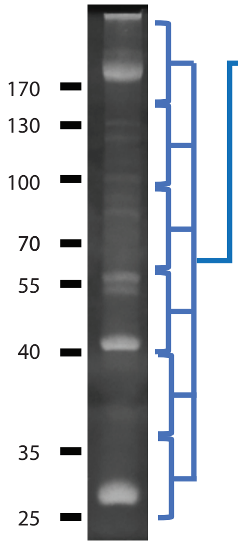

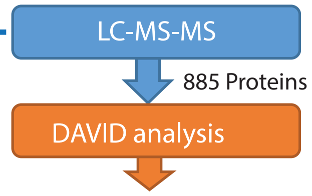

Biological Process	Number of gene
lipid transport	13
lipid homeostasis	7
lipid biosynthetic	21
cholesterol transport	5
cholesterol homeostasis	4
cholesterol biosynthetic	4

lipid transport

UNIPROT_ACCESSION	GENE NAME
27403	ATP-binding cassette, sub-family A (ABC1), member 7(Abca7)
19299	ATP-binding cassette, sub-family D (ALD), member 3(Abcd3)
11982	ATPase, class V, type 10A(Atp10a)
12491	CD36 molecule(Cd36)
14081	acyl-CoA synthetase long-chain family member 1(Acsl1)
11450	adiponectin, C1Q and collagen domain containing(Adipoq)
16952	annexin A1(Anxa1)
12306	annexin A2(Anxa2)
12389	caveolin 1, caveolae protein(Cav1)
11770	fatty acid binding protein 4, adipocyte(Fabp4)
16592	fatty acid binding protein 5, epidermal(Fabp5)
110611	high density lipoprotein (HDL) binding protein(Hdlbp)
12257	translocator protein(Tspo)

lipid homeostasis

UNIPROT_ACCESSION	GENE NAME
13205	DEAD box helicase 3, X-linked(Ddx3x)
12389	caveolin 1, caveolae protein(Cav1)
11770	fatty acid binding protein 4, adipocyte(Fabp4)
19383	hnRNP-associated with lethal yellow(Raly)
56428	mitochondrial carrier 2(Mtch2)
20848	signal transducer and activator of transcription 3(Stat3)
12257	translocator protein(Tspo)

lipid biosynthetic process

UNIPROT_ACCESSION	GENE NAME
67512	1-acylglycerol-3-phosphate O-acyltransferase 2 (lysophosphatidic acid acyltransferase, beta)(Agpat2)
104112	ATP citrate lyase(Acly)
19299	ATP-binding cassette, sub-family D (ALD), member 3(Abcd3)
226144	ER lipid raft associated 1(Erlin1)
244373	ER lipid raft associated 2(Erlin2)
14081	acyl-CoA synthetase long-chain family member 1(Acsl1)
11370	acyl-Coenzyme A dehydrogenase, very long chain(Acadvl)
16952	annexin A1(Anxa1)
56398	calcineurin-like EF hand protein 1(Chp1)
109754	cytochrome b5 reductase 3(Cyb5r3)
13480	dolichol-phosphate (beta-D) mannosyltransferase 1(Dpm1)
16592	fatty acid binding protein 5, epidermal(Fabp5)
14104	fatty acid synthase(Fasn)
15108	hydroxysteroid (17-beta) dehydrogenase 10(Hsd17b10)
56348	hydroxysteroid (17-beta) dehydrogenase 12(Hsd17b12)
223722	malonyl CoA:ACP acyltransferase (mitochondrial)(Mcat)
17918	myosin VA(Myo5a)
28000	pre-mRNA processing factor 19(Prpf19)
18563	pyruvate carboxylase(Pcx)
20655	superoxide dismutase 1, soluble(Sod1)
12257	translocator protein(Tspo)

cholesterol transport

UNIPROT_ACCESSION	GENE NAME
27403	ATP-binding cassette, sub-family A (ABC1), member 7(Abca7)
12491	CD36 molecule(Cd36)
11450	adiponectin, C1Q and collagen domain containing(Adipoq)
12306	annexin A2(Anxa2)
12389	caveolin 1, caveolae protein(Cav1)

cholesterol homeostasis

UNIPROT_ACCESSION	GENE NAME
12389	caveolin 1, caveolae protein(Cav1)
11770	fatty acid binding protein 4, adipocyte(Fabp4)
19383	hnRNP-associated with lethal yellow(Raly)
12257	translocator protein(Tspo)

cholesterol biosynthetic process

UNIPROT_ACCESSION	GENE NAME
226144	ER lipid raft associated 1(Erlin1)
244373	ER lipid raft associated 2(Erlin2)
109754	cytochrome b5 reductase 3(Cyb5r3)
20655	superoxide dismutase 1, soluble(Sod1)

Supplemental Figure 2: Proteomic analyses of stomatin-associated proteins in 3T3-L1 adipocyte-like cells.
Mouse 3T3-L1 cells over-expressing Flag-tagged human stomatin (hSTOM-Flag) were induced to differentiate into adipocyte-like cells and subjected to immunoprecipitation assays using anti-Flag antibody as baits. The pull-down proteins could be separated into 6 fragments by gradient gels, and analyzed after in-gel digestion by LC-MS/MS. A total of 885 potentials "stomatin-interacting proteins" were identified. Proteins that shared significant peptide evidence were classified via using Functional Annotation Tool from DAVID Bioinformatics Resources. Source data are provided as a Source data file.

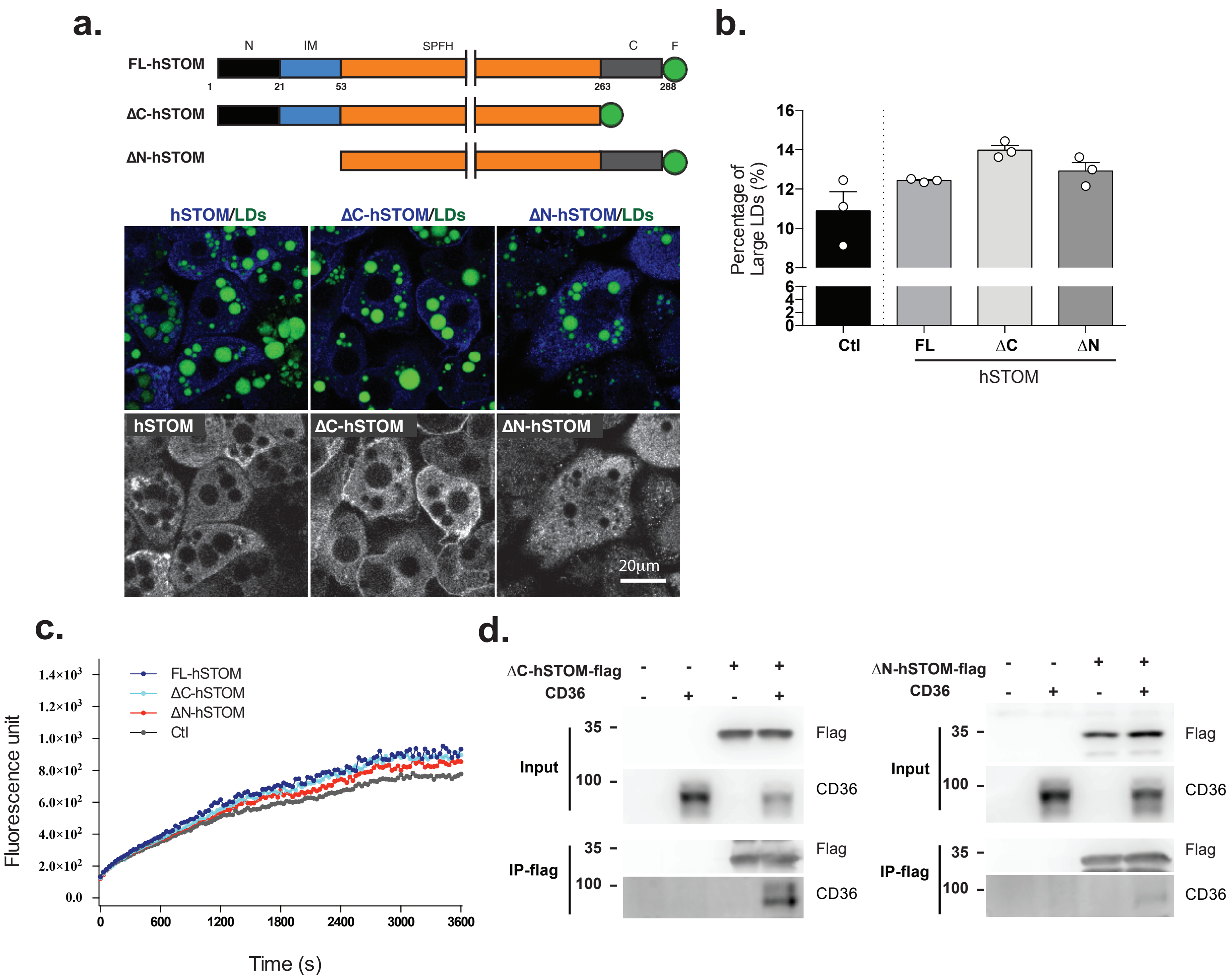

Supplementary Figure 3: Stomatin mutants and their functional characterizations.

a. Flag-tagged full-length human stomatin (FL-hSTOM:1-288aa), C-terminal truncated (ΔC-hSTOM: 1-263aa) and N-terminal truncated (ΔN-hSTOM: 54-288aa) mutants were constructed. Bar = 20 μm. **b.** The numbers and sizes of LD in adipocyte-like cells over-expressing stomatin mutant proteins were measured. Percentages of large LDs (>70 μm^2) among all LDs were calculated. Cells over-expressing FL-hSTOM, ΔC-hSTOM, and ΔN-hSTOM contained larger LDs than the control cells. Mean ± s.e.m. for three independent experiments. **c.** Adipocyte-like cells expressing Flag-tagged FL-hSTOM, ΔC-hSTOM, ΔN-hSTOM, or vector control (Ctl) were treated with 0.2 μM fluorescently-labeled fatty acid (Bodipy-FL-C_{16}) to measure uptake of extracellular fatty acid into the cells. Intracellular accumulations of fluorescence over time were recorded and plotted as a function of time. Each dot shows a mean for three independent experiments. **d.** Cells were co-transfected with CD36 and Flag-conjugated ΔC-hSTOM or ΔN-hSTOM. Total cell lysates were immunoprecipitated with anti-Flag antibody-mediated conjugated beads, and analyzed by immunoblotting assays using antibodies against Flag, or CD36. Source data are provided as a Source data file.

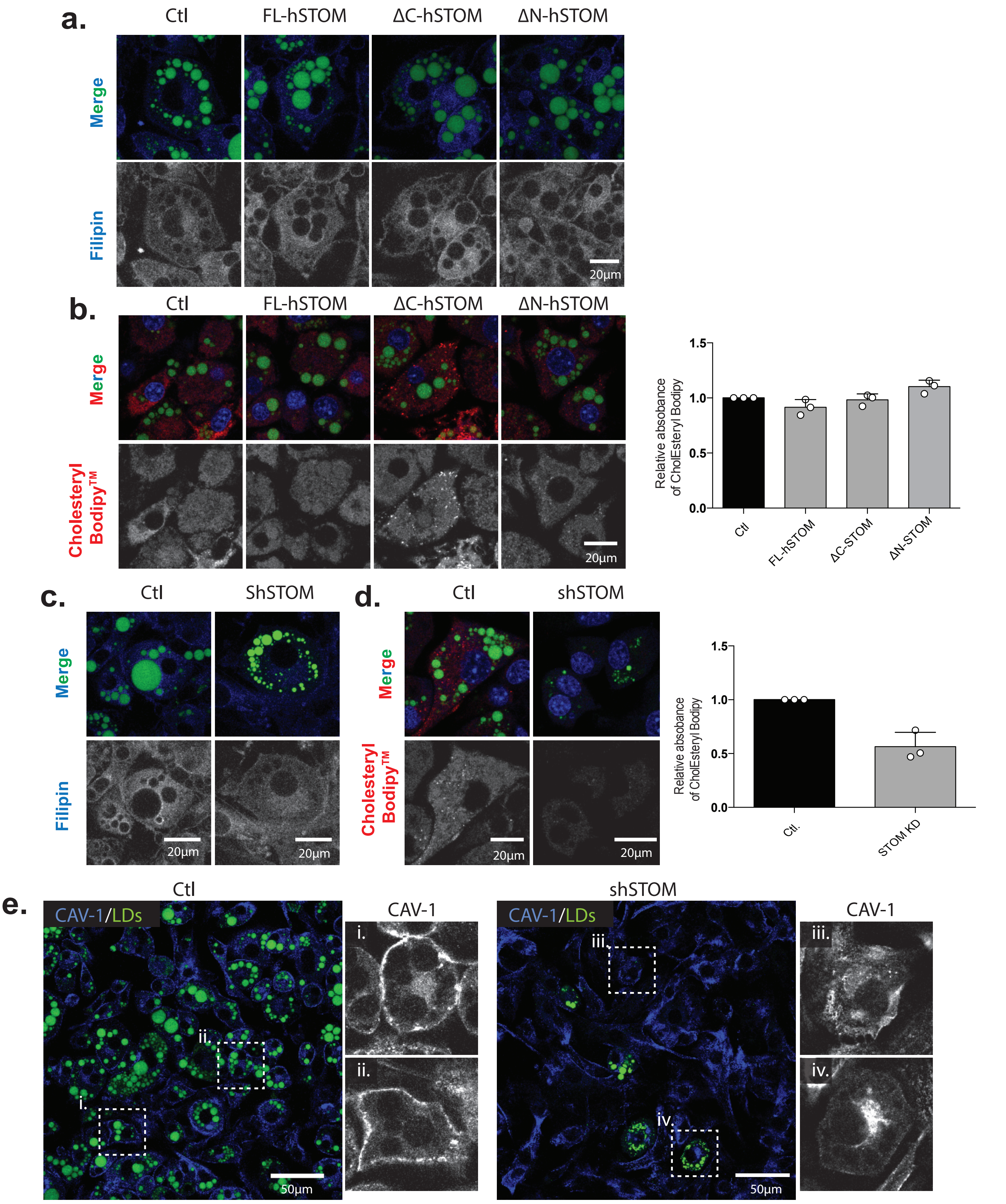

Supplementary Figure 4: Regulations of free cholesterol or caveolin-1 distribution and cholesterol uptake efficiency by stomatin.

a-b. 3T3-L1 adipocyte-like cells were over-expressed with full-length human stomatin (FL-hSTOM), mutants ΔC-hSTOM, or mutant ΔN-hSTOM. LDs were labeled by adding Bodipy-FL to the medium Bar = 20 µm. **a.**The cells were subjected to filipin staining that revealed distribution of free cholesterol inside the cell , or **b.**treated with 1 µM fluorescently-labeled CholEsteryl (CholEsteryl Bodipy 542/563 C_{11}) that measured uptake of extracellular cholesterol into the cell. Mean ± s.d. for three independent experiments. **c-d.** Knockdown of stomatin in 3T3-L1 adipocyte-like cells was done by shRNA method Bar = 20 µm. **c.** Decreased stomatin was noted to down-regulate free cholesterol content of the cell , and **d.** reduce uptake of extracellular cholesterol into the cell. Mean ± s.d. for three independent experiments. **e.** Immunostaining using anti-CAV-1 antibody was done shSTOM adipocyte-like cells and control cells (Ctl). LDs were labeled with Bodipy-FL. Abundant CAV-1 was found on the plasma membrane of the control cells, however, such cell surface presence was reduced by stomatin knockdown (insets). Bar = 50 µm. Source data are provided as a Source data file.

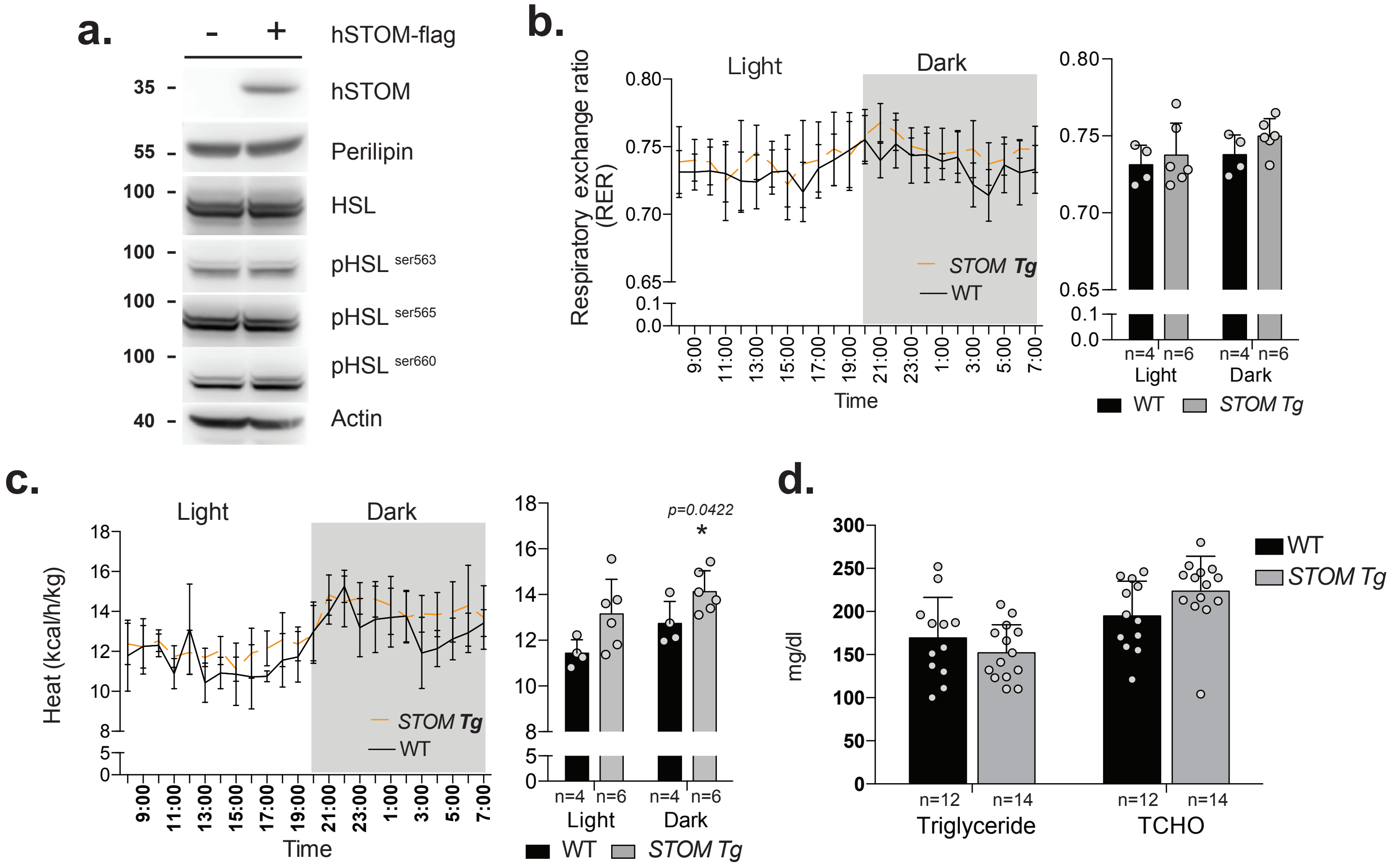

Supplementary Figure 5: Over-expressions of stomatin in mice exhibited little functional impairments in lipid metabolisms

a. The protein amounts of stomatin, perilipin, hormone-sensitive lipase (HSL), and its various phospho-HSL forms (ser^{563}, ser^{565}, and ser^{660}) were analyzed by Western blotting in adipocyte-like 3T3-L1 cells over-expressing human stomatin. There is no significant difference between cells over-expressing stomatin and the control cells. **b.** Energy expenditure and **c.** thermogenesis of *STOM* Tg mice and WT littermate mice, after HFD feeding for 20 weeks, were determined. Both measurements showed no significant difference in respiratory exchange ratio (RER) and heat production comparing *STOM* Tg mice with the controls. Each dot represents one mouse. Mean ± s.d. is shown. *P<0.05 by multiple unpaired t test. **d.** Serum concentrations of triglyceride and cholesterol in HFD-fed *STOM* Tg mice were not significantly different compared to the WT littermates. Each dot represents one mouse. Each dot represents one mouse. Mean ± s.d. is shown. Source data are provided as a Source data file.

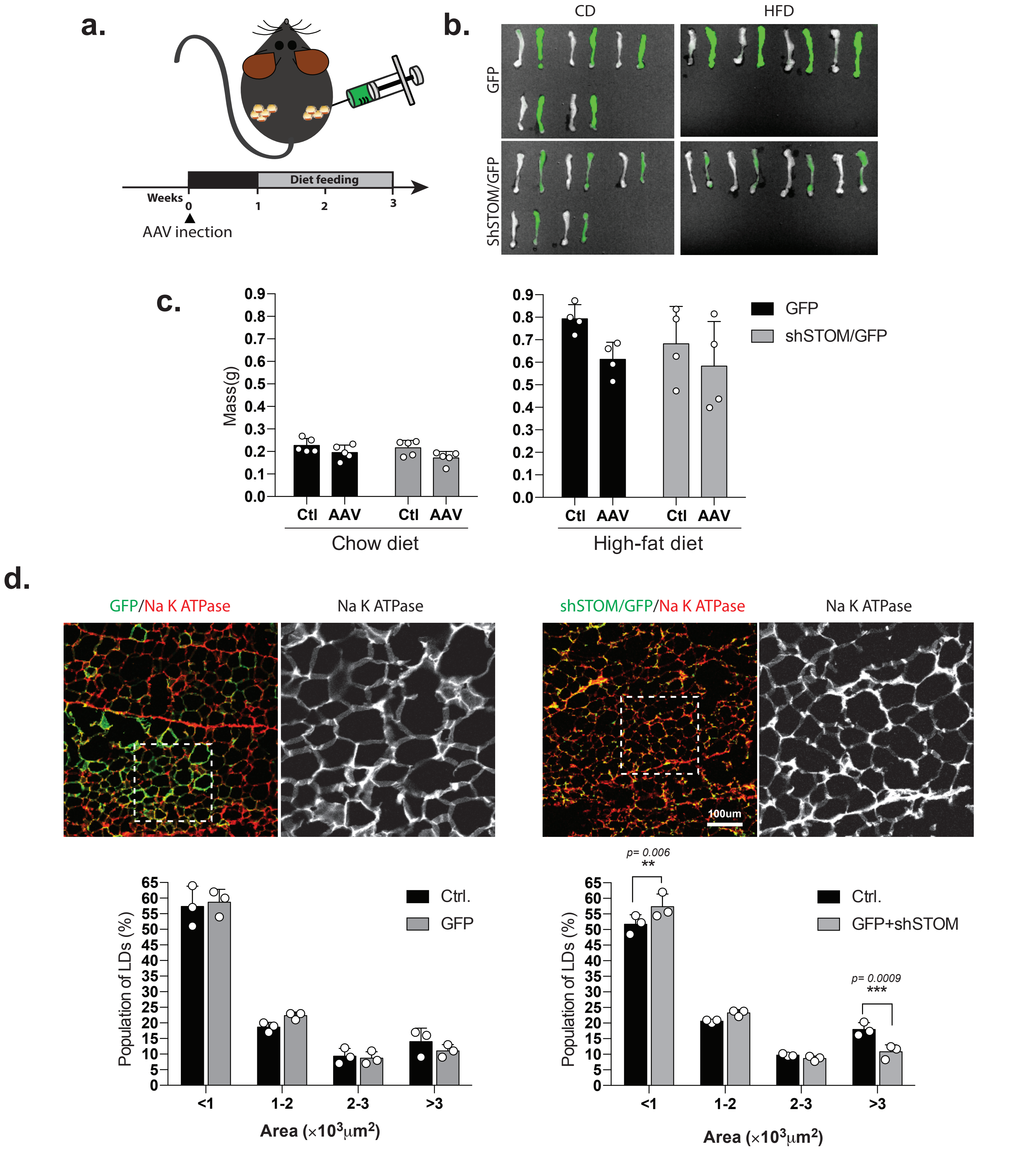

Supplementary Figure 6: Local knockdown of stomatin in subcutaneous adipose tissue reduced high-fat diet-induced adipocytic hypertrophy.

a. AAV virus-carrying shSTOM/GFP knockdown construct or GFP vector alone was injected into a subcutaneous fat pad (SAT). The animals were fed with CD (Mean ± s.d. for five mice /each group) or HFD (Mean ± s.d. for four mice/ each group) for 20 weeks. The GFP signals were used to identify the AAV injection site. The infected SAT pads were dissected, **b.** imaged using Biospace Lab PhotonIMAGER Optima , and **c.** weighed . **d.** Immunofluorescence staining for Na-K-ATPase was employed to identify cell periphery in the tissue section. The size distributions of adipocytes are shown by histogram analyses. Mean ± s.d. for three mice. **P<0.01 and ***P<0.001 by two-way ANOVA analysis. Source data are provided as a Source data file.

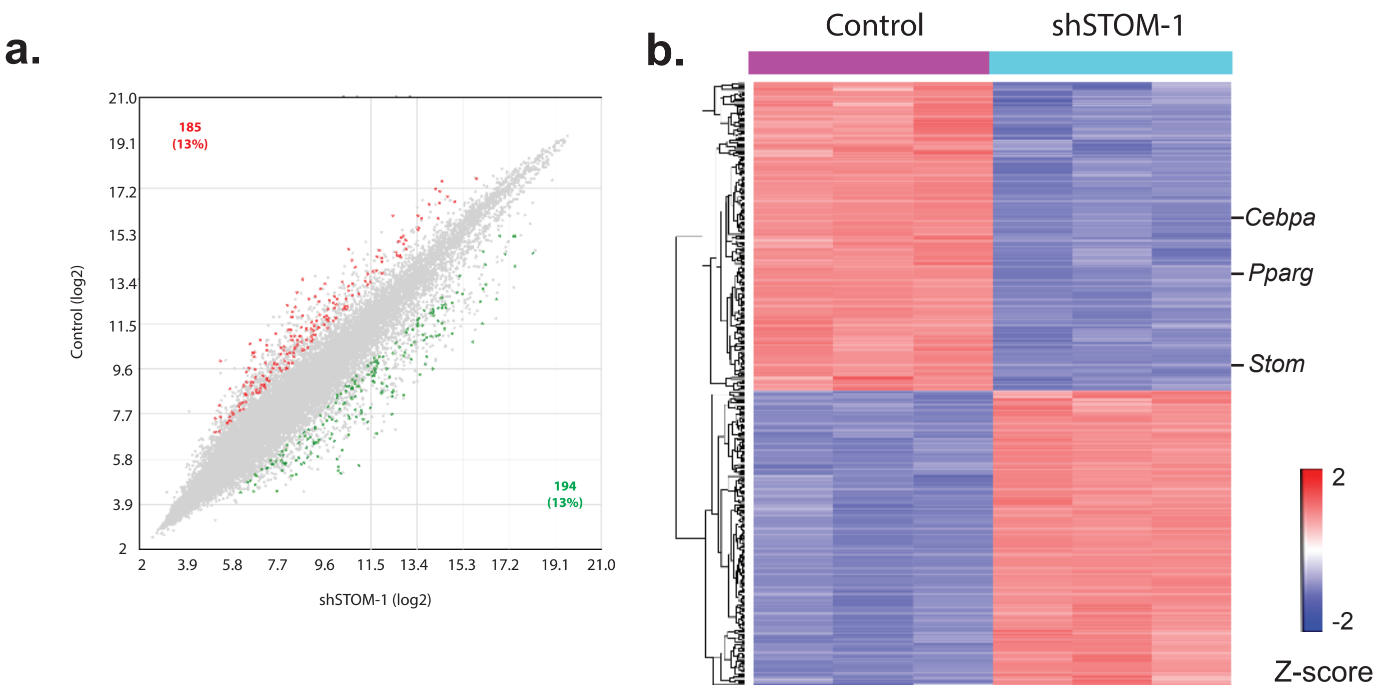

Supplementary Figure 7: Differential expressions of shSTOM-1 and control cells after induction of adipogenic differentiation.

a. Scatter plots of the normalized signal intensities of 1,478 genes that exhibited differential expressions between shSTOM-1 and control cells. Log2 intensities for each spot on the microarray were plotted on the x and y axes with signals from root tips stressed for control and shSTOM-1. The diagonal lines represent fold change cutoffs of ±3. The red spots represent up-regulated genes and the green spots indicate down-regulated genes. **b.** Hierarchical cluster analyses of the 1,478 genes shown in a. Data were collected from three independent cell clones. Relative expression levels of genes are illustrated by the color gradient (Z-score). Source data are provided as a Source data file.

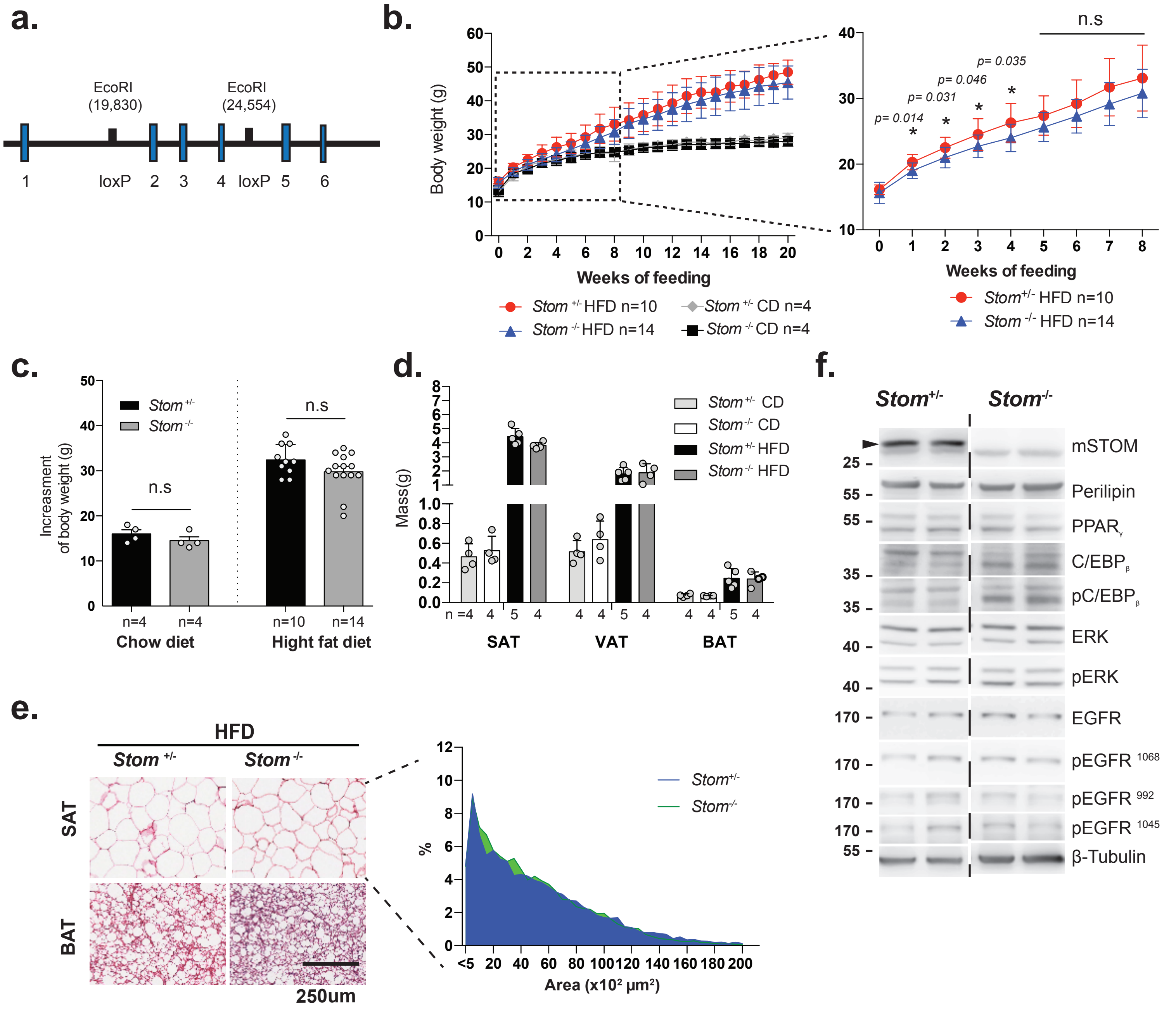

Supplementary Figure 8: Depletion of Stom in mice by knock-out experiments exhibited no discernible phenotype.
a. The Cre-loxP-based chromosome engineering strategy was used here. **b.** Growth curves of Stom$^{-/-}$ mice and Stom$^{+/-}$ littermates fed with regular chow diet (CD) or high-fat diet (HFD) for 20 weeks are shown. There were only transient less bodyweight gains in Stom$^{-/-}$ mice than Stom$^{+/-}$ littermates in the first four weeks of HFD feeding. **c.** No significant difference was noted between knock-out and control mice fed with CD or HFD for 20 weeks in body weight gain. **d.** The masses of SAT, VAT, and BAT were also similar. **c-d** Each dot represents one mouse. Mean ± s.d. of individual mouse. n.s = non-significant, *P<0.05 by multiple unpaired t test analysis. Source data are provided as a Source data file. **e.** Representative H&E stained histopathologic sections of SAT, and BAT prepared from HFD-fed Stom$^{-/-}$ mice and Stom$^{+/-}$ littermates. Histogram analyses also revealed little difference in sizes of adipocytes. Mean ± s.d. for two independent mice. Scale bar = 250 μm. **f.** Western blotting analyses of proteins collected from adipose tissues of Stom$^{-/-}$ and Stom$^{+/-}$ littermate mice. Source data are provided as a Source data file.

Supplementary Table 1: Primer list for qPCR assay

Gene	Species	Forward	Revered
Pparg	Mus musculus	5'AGGCCGAGAAGGAGAAGCTGTTG3'	5'TGGCCACCTCTTTGCTCTGCTC3'
Cebpa	Mus musculus	5'GACATCAGCGCCTACATCGA3'	5'TCGGCTGTGCTGGAAGAG3'
Cebpb	Mus musculus	5'ATTTCTATGAGAAAAGAGGCGTATGT3'	5'AAATGTCTTCACTTTAATGCTCGAA3'
Cebpd	Mus musculus	5'TTCCAACCCCTTCCCTGAT3'	5'CTGGAGGGTTTGTGTTTTCTGT3'
Stom	Mus musculus	5'GTGCACTGACAGCCTCATCAA3'	5'AGCGCATTCCTGAGGGTAGTT3'
Nono	Mus musculus	5'TGCTCCTGTGCCACCTGGTACTC3'	5'CCGGAGCTGGACGGTTGAATGC3'
Dlk1	Mus musculus	5'ACAATGTCTGCAGGTGCCAT3'	5'AGGTCCACGCAAGTTCCATT3'
Fabp4	Mus musculus	5'TGAAATCACCGCAGACGACAGG3'	5'GCTTGTCACCATCTCGTTTTCTC3'
Cfd	Mus musculus	5'ACCTGACAGCCTTGAGGACGAC3'	5'GGGTTCCACTTCTTTGTCCTCG3'
