# Supplementary material for: Stomatin modulates adipogenesis through the ERK pathway and regulates fatty acid uptake and lipid droplet growth
Source: Nat Commun. 2022 Jul 19;13:4174. doi: 10.1038/s41467-022-31825-z (PMC9296665; doi:10.1038/s41467-022-31825-z)
Supplement: Supplementary file 3 — Description of Additional Supplementary Files [file 41467_2022_31825_MOESM3_ESM.pdf]

Description of Additional Supplementary Files

File name: Supplementary Data 1

Description: The dataset of stomatin-associated proteins was identified by a Liquid Chromatography with Tandem Mass Spectrometry-Based Proteomic assay (LC-MS-MS).

File name: Supplementary Movie 1

Description: The time-lapse recording under phase contrast and fluorescence microscopy of the sequence of a LD-LD fusion event within a live adipocyte-like 3T3-L1 cell transfected with hSTOM-RFP. Time is shown in hr : min.
