# Supplementary material for: Stomatin modulates adipogenesis through the ERK pathway and regulates fatty acid uptake and lipid droplet growth
Source: Nat Commun. 2022 Jul 19;13:4174. doi: 10.1038/s41467-022-31825-z (PMC9296665; doi:10.1038/s41467-022-31825-z)
Supplement: Supplementary file 6 — Reporting Summary [file 41467_2022_31825_MOESM6_ESM.pdf]

nature portfolio

NCOMMS-20-42019B
Corresponding author(s): Chi-Hung Lin
Last updated by author(s): Jun 1, 2022

Reporting Summary

Nature Portfolio wishes to improve the reproducibility of the work that we publish. This form provides structure for consistency and transparency in reporting. For further information on Nature Portfolio policies, see our Editorial Policies and the Editorial Policy Checklist.

Statistics

For all statistical analyses, confirm that the following items are present in the figure legend, table legend, main text, or Methods section.

n/a	Confirmed	
☐	☒	The exact sample size (*n*) for each experimental group/condition, given as a discrete number and unit of measurement
☐	☒	A statement on whether measurements were taken from distinct samples or whether the same sample was measured repeatedly
☐	☒	The statistical test(s) used AND whether they are one- or two-sided<br>*Only common tests should be described solely by name; describe more complex techniques in the Methods section.*
☒	☐	A description of all covariates tested
☐	☒	A description of any assumptions or corrections, such as tests of normality and adjustment for multiple comparisons
☐	☒	A full description of the statistical parameters including central tendency (e.g. means) or other basic estimates (e.g. regression coefficient) AND variation (e.g. standard deviation) or associated estimates of uncertainty (e.g. confidence intervals)
☐	☒	For null hypothesis testing, the test statistic (e.g. *F*, *t*, *r*) with confidence intervals, effect sizes, degrees of freedom and *P* value noted<br>*Give P values as exact values whenever suitable.*
☒	☐	For Bayesian analysis, information on the choice of priors and Markov chain Monte Carlo settings
☒	☐	For hierarchical and complex designs, identification of the appropriate level for tests and full reporting of outcomes
☒	☐	Estimates of effect sizes (e.g. Cohen's *d*, Pearson's *r*), indicating how they were calculated

Our web collection on statistics for biologists contains articles on many of the points above.

Software and code

Policy information about availability of computer code

Data collection	ImageQuant LAS4000 (GE Healthcare Life Science), i-control 1.11, Magellan 7, Zen software 2011 SP7 (Zeiss), StepOne software (Applied Biosystems), Aperio CS2 Digital Pathology Scanner (Leica), Metamorph (Version 7.8.8.0), GeneChip expression console
Data analysis	Transcriptome Analysis Console (TAC4.0), ImageJ 1.48V, GraphPad Prism7, Excell 2016, Aperio ImageScope(v12.3.2.8013), Fiji Adiposoft software, Metamorph (Version 7.8.8.0), Magellan 7, Zen software 2011 SP7 (Zeiss), PEAKS Studio 8.5 (Bioinformatic silution, Ontario,CA)

For manuscripts utilizing custom algorithms or software that are central to the research but not yet described in published literature, software must be made available to editors and reviewers. We strongly encourage code deposition in a community repository (e.g. GitHub). See the Nature Portfolio guidelines for submitting code & software for further information.

Data

Policy information about availability of data

All manuscripts must include a data availability statement. This statement should provide the following information, where applicable:

- Accession codes, unique identifiers, or web links for publicly available datasets
- A description of any restrictions on data availability
- For clinical datasets or third party data, please ensure that the statement adheres to our policy

All data supporting the finding of this study are available within this paper and its supplementary information files.The expression profiling microarray data has been deposited in public Gene Expression Omnibus (GEO) database under the accession codes GSE205011. The mass spectrometry proteomics data have been deposited to the ProteomeXchange Consortium via the PRIDE 72 partner repository with the dataset identifier PXD031866 and 10.6019/PXD031866. Source data are provided with this paper.

Field-specific reporting

Please select the one below that is the best fit for your research. If you are not sure, read the appropriate sections before making your selection.

- [x] Life sciences
- [ ] Behavioural & social sciences
- [ ] Ecological, evolutionary & environmental sciences

For a reference copy of the document with all sections, see nature.com/documents/nr-reporting-summary-flat.pdf

Life sciences study design

All studies must disclose on these points even when the disclosure is negative.

Sample size	No statistical method was used to pre-determine the sample size.
Data exclusions	The sick, injured, and deformed mice were excluded from the analysis.
Replication	The experimental results were reproduced, and the replicated numbers are described in figure legends.
Randomization	Animals were randomly allocated for each group.
Blinding	In vivo lipid uptake assay was performed by an outside lab blinded to test.<br>The mouse metabolism and body composition related experiments were blinded examined by Taiwan Mouse Clinic from the Academia Sinica.<br>Blinding was not relevant in cellular experiments since we need to compare the different gene-modified cells.

Reporting for specific materials, systems and methods

We require information from authors about some types of materials, experimental systems and methods used in many studies. Here, indicate whether each material, system or method listed is relevant to your study. If you are not sure if a list item applies to your research, read the appropriate section before selecting a response.

Materials & experimental systems

n/a	Involved in the study
[ ]	[x] Antibodies
[ ]	[x] Eukaryotic cell lines
[x]	[ ] Palaeontology and archaeology
[ ]	[x] Animals and other organisms
[x]	[ ] Human research participants
[x]	[ ] Clinical data
[x]	[ ] Dual use research of concern

Methods

n/a	Involved in the study
[x]	[ ] ChIP-seq
[x]	[ ] Flow cytometry
[x]	[ ] MRI-based neuroimaging

Antibodies

Antibodies used

Primary antibodies:
Perilipin (#9349), PPAR gamma(#2435), C/EBP alpha (#8178), pERK (#4370), ERK (#4965), pAkt (#4060), Akt (#4691), lipolysis Activation Antibody sampler kit (#8334), hCD36 (#14347) and ATP1A1(#3010) from Cell Signalling.
STOM(a166623b) from Abcam.
beta-Tubulin(T5201) from Sigma Aldrich.
Flag (CSB-MA000021M0m) from Cusabio.
mCD36 (820263-T48) from SinoBiological.
beta-Actin (GTX109639) , Perilipin (GTX2781) from GeneTex.
Human STOM (M14), Human STOM(E6), CAV-1 (7C8), CAV-1 (N20), CD36 (SM), GFP (FL) from Santa Cruz Biotechnology.
AF647-632 mCD36 (MF3) from Bio-Rad.

Secondary antibodies:
Alexa Fluor® 647 AffiniPure Goat Anti-Rabbit IgG (H+L)(#111-605-003), Rhodamine Red™-X (RRX) AffiniPure Goat Anti-Mouse IgG (H+L)(#115-545-003), Rhodamine (TRITC) AffiniPure Goat Anti-Rabbit IgG (H+L)(#111-025-144) and Peroxidase conjugated Goat anti-Mouse IgG (H+L) (#115-035-003), Peroxidase conjugated Goat anti-Rat IgG Fc Fragment specific (#112-035-071) from jackson ImmunoResearch.
Peroxidase conjugated Goat anti-Rabbit IgG (H+L) (Ap132P) from Chemicon®
Peroxidase conjugated Donkey anti-Goat IgG (H+L) (sc-2020) from Santa Cruz Biotechnology.
VeriBlot for IP Detection Reagent (HRP) (ab131366) from Abcam.

Validation

All primary antibodies used in this study were validated by the manufacturer company. The information of its validation data or

citation can be found on the manufacturer website by searching the catalog number of antibodies provided in the materials and methods section.

Eukaryotic cell lines

Policy information about cell lines

Cell line source(s)	3T3-L1 cell line was obtained from BCRC.<br>HEK-293T cell line was obtained from ATCC.
Authentication	None of the cell lines used were authentication
Mycoplasma contamination	All cell lines were confirmed to be negative for mycoplasma contamination by the EZ-PCR™ Mycoplasma Detection Kit (Biological Industries) or hochest staining.
Commonly misidentified lines<br>(See ICLAC register)	None

Animals and other organisms

Policy information about studies involving animals; ARRIVE guidelines recommended for reporting animal research

Laboratory animals	STOM transgenic and STOM knockout mice were generated and described in the "Method" section. 3-4 weeks male STOM Tg, STOM KO, and wild-type littermates were fed with either a chow diet or a high-fat diet (D12492, 60 kcal% fat, Research Diets) under free-feeding conditions. All of the mice were housed on a 12-hr light/dark cycle at 22°C.
Wild animals	No wild animals were used in this study
Field-collected samples	No field-collected samples were used in this study
Ethics oversight	All the animals experiments were approved by Institutional Animal Care and Use Committee (IACUC) of National Yang Ming Chio Tung University.(IACUC #1050801, 1050801r,1090706,1090706r)

Note that full information on the approval of the study protocol must also be provided in the manuscript.
